# Supplementary material for: Are carbon emissions trading and green financial instruments synergistic? -Comprehensive quantitative research based on content analysis
Source: PLoS One. 2024 Mar 7;19(3):e0298601. doi: 10.1371/journal.pone.0298601 (PMC10919601; doi:10.1371/journal.pone.0298601)
Supplement: S1 Data — (ZIP) [file pone.0298601.s001.zip › Data/地级市Z_F工作报告.zip!/║┌┴·╜¡/┤≤╟∞╩╨/└┤╫╘╣1⁄2╓┌║┼í╛┬φ┐╦╩2╛▌═°í┐_2007.docx]

各位代表：

　　现在，我代表大庆市人民政府，向大会作工作报告，请予审议，并请政协委员提出意见。

　　一、2006年和过去四年工作回顾

　　2006年是实施“十一五”规划的起步之年。我们紧紧围绕市委确定的“创建百年油田、构建战略新高、共建和谐社会，推动大庆迈上科学发展新阶段”这一主题，积极进取，务实拼搏，全力推进56件大事实事和48项重点工作，圆满完成了政府工作的主要目标和任务，实现了“十一五”良好开局。

　　———国民经济持续快速增长。

　　全市地区生产总值1620.3亿元，增长10.5%。地方财政总收入84.8亿元，增长21.7%。固定资产投资384.6亿元，增长27.2%。农业和农村经济健康发展，实现增加值50.3亿元，增长11.5%。粮食总产25.75亿公斤，增长8.6%，达到历史最好水平；畜牧业实现增加值25亿元，增长13%，大庆成为全国牛奶生产10强市之一。工业经济持续增长，实现增加值1352亿元，增长9.6%。生产原油4340万吨、天然气24.5亿立方米，石油工业实现增加值1101亿元，增长4.7%；20万吨醋酸、10万吨干气制乙苯等大项目进展顺利，规模以上石化工业实现增加值90亿元，增长16.3%；地方工业持续高速增长，新建续建亿元以上项目34个，实现增加值153亿元，增长37%。高新区快速发展，实现工业增加值71亿元，占地方工业的46.4%。启动西城工业园，完成4平方公里基础设施建设，具备企业入驻条件。商贸、金融、旅游等第三产业实现增加值181.4亿元，增长16.3%。社会消费品零售总额达到252.2亿元，增长16.3%。非公有制经济实现增加值251亿元，增长20%。大庆进入2006年度全国“完美假期”十佳精品旅游线路。

　　———新农村建设开局良好。

　　建设抗旱保收田74万亩，特色绿色基地面积达到300万亩；建设通乡村公路937公里，在全省提前4年实现“村村通”；建成饮水安全工程90处，新组建农机合作社49个，实施节能康居、清洁能源利用、有线电视村村通等工程，农民生产生活条件得到改善。全面完成中小学D级危房改造，59所农村寄宿制学校全部实现集中供暖，农村学校远程教育在全省率先实现“校校通”，共有10.2万人次得到“两免一补”政策救助；推行农村新型合作医疗，农村常住人口参合率达到88%，筹资总额、覆盖面、参合率、政府补贴和个人筹资五项指标全省最高。发放“一免两补”等政策性补助1.8亿元，转移农村劳动力22万人，实现劳务收入14亿元。发展标准化农资店和配送中心471家，大庆被列为国家“万村千乡”市场工程20个试点城市之一。恢复建设农村科技、文化设施，农村文化阵地建设加强。帮扶共建成效明显，市直部门和大企业落实帮扶资金9720万元，帮建项目246个。

　　———人民生活质量继续提高。

　　城镇居民人均可支配收入实现14791元，农民人均纯收入实现3610元，分别增长8.3%和12.7%。居民储蓄存款余额573.4亿元，增长8.9%。就业方面，全市新增就业3.1万人，市区“零就业”家庭实现每户至少有一人就业。保障方面，城镇“五险”参保人数新增8.6万人次，解决拖欠农民工工资2088万元，市区职工最低工资标准由390元调整到620元，农村57所敬老院改造合并工程全部结束。救助方面，城镇居民最低生活保障标准由195元提高到247元，覆盖人群6.9万人。实施城市特困群体大病救助，供热救助9963户。启动农村居民最低生活保障制度，7万人纳入低保。减负方面，扩大居民燃气补贴范围，6.8万户受益。城市物业服务收费标准每平方米减少4.6元。创建“放心医院”和“诚信平价药店”，市级医院人均住院费下降192元，100种常用药品价格与国家定价相比平均降低35.8%。完成5.6万户已建住宅民用天然气入户工程。企业退休人员养老金月增加135元，机关事业单位职工收入月增加526元，离退休人员月增资395元。公安消防“110”、“119”、“122”三台合一，“96760”社区服务中心启动运行，群众生活更加方便。

　　———城市建设跨越发展。

　　续建新建项目565项，301国道绕行线、中三路改造、滨洲线“平改立”和中央大街立交桥等5路12桥相继竣工，萨尔图机场启动建设。铁人王进喜纪念馆、油田历史陈列馆建成开放，行政服务中心、会议中心、教育文化中心和青少年科技活动中心等功能项目完成主体工程。开发建设新区住宅378万平方米，五湖新区、红旗地区落成一批中高档住宅。提升城市管理水平，拆除违法违章建筑17万平方米，清理占压油气管线建筑5.7万平方米，改造府明、万宝等老居住区32个，完成红旗村改造二期工程，东城区实施城市数字化管理。城市经营成效明显，土地经营收益26.7亿元，协调落实城建资金14亿元。投资30亿元改善城市生态环境，治理湖泡3个，启动建设生态园7个，新建城市绿地585万平方米，植树造林15万亩。

　　———社会秩序安定和谐。

　　实施“四化一畅”工程，强化城市环境综合治理，查处销毁“八种”违法车辆2526台；集中清理和整顿建筑市场，45户违规建筑企业资质降级、吊销或清出大庆建筑市场；开展市场专项治理，查获黑窝点87处，收缴销毁假冒伪劣产品价值130万元；平抑农资、医疗等市场价格，城市环境和管理秩序继续改善，大庆被公安部评为A类畅通城市。加强“平安大庆”建设，强化警民联防、电子监控等治安防范措施，严厉打击各种违法犯罪。开展打击非法取水专项治理活动，封堵水井768眼，减少地下水开采量1000万立方米。加大环保执法力度，关停整顿环保违规企业26家。深入推进重点行业和区域安全生产专项治理，安全事故比上年下降7.4%，伤亡人数分别下降2.8%和2.7%。重视群众来信来访，市政府领导信访接待日接待群众450人次，市长热线电话受理群众来电9.7万件、处理率达到95%。

　　———公共事业全面进步。

　　大力发展教育事业，新建滨洲和新潮学校，改造大庆中学等52所学校，高考万人升学率连续四年位居全省首位，在校大学生达到6.2万人，职业教育培养中高等专业人才2.8万人。提高医疗卫生服务水平，建设形成眼病、骨创伤治疗等特色专科。全省首家推行医疗机构之间辅助检查相互认可等10项措施，实行药品集中招标采购，减轻患者负担9200万元。科技创新能力不断增强，引进省农科院大庆分院等11家科研院所，成功申报1个国家级、6个省级工程技术中心，“大庆外围油田年产500万吨原油开发技术应用研究”获国家科技进步二等奖。文体事业蓬勃发展，“书香大庆”、“大庆之冬”、“激情之夏”等群众性文体科普活动广泛开展。竞技体育长足进步，夺得多哈亚运会女子举重金牌一枚。大庆跻身2006年度“中国十佳魅力城市”。

　　———改革开放步伐加快。

　　深化公安警务体制改革，一线办案警力超过85%，防范和打击能力显著提高。推行物业管理体制改革，实行服务分等收费，组建社区业主委员会235个，物业管理水平得到提升。研究事业单位改革有关政策，准备工作已经完成。积极推进广电集团体制改革，实现管办分离、政事分开。继续完善集中收付、投资评审和政府采购制度，规范财政资金管理。高新区全员竞聘上岗，实现体制机制转换。推进招商引资“五个转变”，引进项目655个，其中亿元以上项目34个，全市实际到位资金105亿元。外经外贸发展迅猛，进出口贸易总额实现4.4亿美元，实际利用外资4429万美元，分别增长61.8%和43.8%。银螺、碧港、汉维长垣3户企业在海外成功上市。华科公司股权分置改革顺利完成，联谊公司与深圳淞江投资担保公司成功重组，企业获新生机。

　　———政府自身建设加强。

　　坚持科学、依法和民主行政，出台决策程序、行政问责、责任追究等制度，建立项目审批、招标采购、资金使用等管理程序，推进议事规则和制度体系建设；认真落实市人大及其常委会决议、决定，重视支持人民政协履行职能，办理人大代表建议212件、政协委员提案176件；听取吸纳社会各界意见，征集人民建议353件，规范政府施政行为。深入推进政务公开，依法确定市政府行政权力122项、部门行政权力2033项，全部向社会公开。加强政府廉政建设，查办商业贿赂案件47宗，强化行政效能监察，机关工作效率提高。

　　此外，工商税务、邮政通讯、新闻出版、民族宗教、文史档案、外事侨务、审计统计、海关商检、国防人防、计划生育等项工作，也取得显著成绩。

　　各位代表，本届政府任期已满。回首过去四年工作，我们以发展经济、改善民生为主线，抓住大事，办好实事，破解难事，大庆可持续发展取得了新成就。

　　过去四年，是坚持科学发展，转变经济增长方式，实现经济又好又快发展的四年。地区生产总值年均实现两位数增长，是建市以来增长最快时期。累计生产原油1.8亿吨、天然气90亿立方米，油与非油经济比例由75:25调整到64.6:35.4，非公有制经济在全市经济中的比重由12.4%提高到15.5%。单位生产总值能源消耗年均降低4.2%，高新技术产业产值年均递增28%。大庆在全国百强城市综合实力评比中居于前列，人均地区生产总值在全国地级以上城市中位居第14位。

　　过去四年，是坚持以人为本，着力维护群众利益，人民得到较多实惠的四年。新增就业15.9万人次，城市养老、失业保险金发放率和低保覆盖率达到100%，率先在全省启动农村低保，城镇低保标准全省领先。实施环境再造、富民安居工程，改造棚户区87.8万平方米，建设外来人口居住区14万平方米。妥善解决市政会战家属待遇、原三永村和乙烯地区征地、铁西动迁等历史遗留问题。城乡居民收入比由5.4:1缩小到4.1∶1。大庆在中国城市生活质量排行榜中名列第22位，位居东北城市中第2位。

　　过去四年，是打造现代都市框架，东西城区协调发展，城乡面貌发生巨大变化的四年。从开发建设“五湖新区”到外移301国道绕行线，城市空间进一步拓展；从改造红旗村到架桥修路解决交通瓶颈，西城区面貌明显改观。建设城市重大功能项目58个，开发现代居民新区13个，城市品位整体提升。以新农村建设为统领，建设通县乡村公路3284公里，村容村貌发生明显变化。推进生态市建设，植树造林80.4万亩，治理城区大型湖泡8个，新增城市绿地2600多公顷，全面实行草原禁牧，“绿色油化之都、天然百湖之城、北国温泉之乡”品牌在国内外产生影响。

　　过去四年，是致力战略合作，地企关系和谐融洽，区域经济快速发展的四年。地方始终把为石油石化大企业搞好服务放在首位，大企业始终站在全局思考问题，既承担经济责任又承担社会责任。地企双方确立了“和谐双赢”的理念，建立了“大事共商”的机制，共同向上争取30万吨聚丙烯新建、乙烯扩建到60万吨等一批国家级大项目。规范油田征地补偿机制，企业、乡镇、农民三方受益。联合开发外围小油田、治理生态环境、拆除占压油气管线建筑，大企业累计投入城市基础设施建设等方面资金32.9亿元。大庆被国家确定为石油资源型城市可持续发展试点城市，温家宝总理亲临大庆现场办公，研究解决大庆长久繁荣问题。

　　过去四年，是改革开放步伐加快，招商引资连年攀升，经济发展活力明显增强的四年。支持大企业分离办社会职能，推动地方企业改制重组，深化农村税费改革，推进事业单位改革，体制机制不断创新，一些制约经济社会发展的深层矛盾得到有效解决。全面加强对外交流合作，引进亿元以上项目143个、到位资金364.4亿元，外贸进出口总额四年翻三番。大庆被《福布斯》评为中国内地最适合开办工厂的10个城市之一。

　　过去四年，是构建和谐社会，社会事业长足进步，群众物质文化生活不断丰富的四年。全力打造教育名市，引进建设7所高校和91个科研院所，创建省级示范高中10所，高校拥有量和基础教育质量位于全省前列。医疗服务质量和水平不断提高，三级医院领办社区卫生模式在全国推广，疾病预防和救助网络覆盖城乡。组建生产力促进中心，建立企业研发中心，科技创新能力不断增强。社区建设成效明显，被国家确定为21个示范城市之一。国防动员工作取得突出成绩，大庆第四次被评为全国双拥模范城。创作《铁人轶事》等一批文化精品，群众精神文化生活不断丰富。大庆成为全国文明城市创建工作先进市。

　　各位代表，2006年和过去四年所取得的成就，主要得益于市委统揽全局，科学决策；得益于人大、政协履行职责，监督支持；得益于驻庆中省直单位和社会各界密切配合，鼎力相助；得益于全市人民紧密团结，艰苦奋斗；还得益于历届政府和老领导打下的良好工作基础。在此，我代表市政府，向所有为大庆经济发展和社会进步做出贡献的同志们，表示崇高的敬意和衷心的感谢！

　　回顾过去四年成就，我们深刻体会到，政府工作必须坚持以科学发展观为统领，立足创建百年油田、构建战略新高，注重提高经济增长的速度、质量和效益；必须坚持把争取和落实上级政策与解决大庆实际问题紧密结合，注重发展壮大接续产业；必须坚持推进改革扩大开放，注重营造适应市场经济发展、和谐社会建设要求的体制环境；必须坚持关注和改善民生，注重解决群众最关心、最具体、最现实的问题；必须坚持统筹经济社会协调发展，注重加强市场监管、社会管理和公共服务；必须坚持转变政府职能，注重改进工作作风、提高行政效能；必须坚持责任共担、发展共促、稳定共抓、城乡共建，注重地企和谐合作，共同推动大庆的可持续发展。

　　我们虽然取得一定成绩，但还面临一些突出问题和困难，主要是：地方产业中支撑发展的大项目较少，缺少知名企业和名牌产品；非油化企业自主创新能力不强，产业链条较短，经济增长方式相对粗放；农业基础比较薄弱，促进农民增收的长效机制尚未形成；招商引资层次不高，经济外向度较低；现代服务业发展滞后，产业协同关联不紧，对经济增长的贡献率相对较小；城乡部分群众生产生活困难，就业压力较大；一些政府部门和公务人员服务意识不强，政府公共服务水平有待提高，等等。这些问题，都需要在今后的工作中认真解决。

　　二、今后五年发展目标任务和2007年重点工作

　　今后五年，我们要坚持以邓小平理论和“三个代表”重要思想为指导，以科学发展观统领经济社会发展全局，全面落实市七次党代会总体部署，紧紧围绕“创建百年油田，构建战略新高，共建和谐社会，推动大庆迈上科学发展新阶段”的发展思路，构筑以“两城、四区、五极”为重点的总体发展格局，全力发展经济，致力改善民生，着力促进和谐，抢抓历史机遇，力攀战略新高，努力实现大庆可持续发展新跨越。到2011年全市经济社会发展预期目标是：地区生产总值达到2160亿元，年均增长8%以上，非油经济比重达到50%左右。单位生产总值能源消耗比2006年降低20%，科技对经济增长贡献率达到50%以上。城镇登记失业率控制在4.5%以内，人口自然增长率控制在5.6‰以内，城镇居民人均可支配收入达到19000元，农民人均纯收入达到5500元，城镇居民恩格尔系数保持在30%左右。

　　未来五年的主要任务是：

　　———发展石油石化产业要步入新阶段。

　　合力创建百年油田，增加油气后备储量，加快天然气开发，力争到2011年，油气当量保持在4000万吨以上。努力打造千亿元石化产业，新上一批石化大项目，建成以百万吨级乙烯、聚丙烯、甲醇、复合肥、大型芳烃装置为主要标志的世界级石化产业基地，到2011年石化产业实现销售收入1300亿元以上。

　　———壮大地方工业要走出新路子。

　　坚持园区化发展方向，着力打造东部高新技术产业聚集区、西部装备制造业聚集区、南部轻工业聚集区，推进主导产业集群化、经济结构合理化、增长方式集约化，形成乳业、农产品加工、纺织和皮革、机械制造和电子信息等6个超百亿规模的产业集群。到2011年，地方工业力争实现销售收入1100亿元以上，成为支撑全市工业的三大支柱之一。

　　———建设社会主义新农村要迈出新步伐。

　　坚持多予、少取、放活方针，发展现代农业，增加农民收入，不断改变农村面貌。扩大种植业和畜牧业基地规模，扶持壮大龙头企业，进一步提高农业产业化水平。加强基础设施建设，抗旱保收田面积达到600万亩，农机合作社发展到200个，通屯路面硬化率达到90%。在农村推广以户用沼气为主的新型能源，完成安全饮用水入户工程，数字电视入户率达到80%以上。着眼搞活流通和提高农民生活质量，进一步畅通工业品下乡和农产品进城的通道。

　　———服务业发展要跃上新台阶。

　　以东西城区为重点，推进现代服务业扩规模、上水平。引导企业运用现代信息技术，创新流通方式，促进商贸等传统服务业提档升级；培育金融保险业、信息咨询业，壮大现代物流业；依托萨尔图飞机场、北国之春温泉城、春雷软件园，重点发展温泉旅游、空港经济等高层次服务业，打造北部现代服务业聚集区。

　　———解决群众切身利益问题要取得新成效。

　　着力改善民生，让群众在发展中得到更多实惠。千方百计增加就业，新增就业岗位13万个以上。扩大城乡社保覆盖范围，拓展“七位一体”综合救助内容，城镇社保覆盖面达到90%以上，农村低保覆盖率达到100%，农村新型合作医疗参合率达到92%。改善城乡居民居住环境，完成主城区城中村和平房区动迁改造。整治土地征用、房屋拆迁中的侵权行为，治理教育、医疗等行业中存在的损害群众利益问题。

　　———城市基础设施建设水平要有新提升。

　　加快航空、铁路、公路、水运等重大项目建设，完成飞机场、铁路大庆西站、公路客运总站等交通枢纽工程，建设大庆至广州高速公路大庆段、城市轨道交通一期工程，构建现代立体交通体系；搞好教育、医疗、文化、体育等服务设施建设，全面提升城市品位；加强供电、供水、供热、供气、通讯等配套设施建设，不断提高城市承载能力，努力打造现代都市。

　　———改革开放要实现新突破。

　　坚持以改革促开放，以开放促发展，努力形成更具活力、更适应科学发展需要的体制机制环境。深化行政管理体制改革，推进事业单位改革，支持中直石油石化企业深化改革，加快财政、金融、投资、文化等体制改革。大力发展非公有制经济，到2011年占全市经济比重达到20%。加大招商引资力度，着力引进“大高外”项目，在引进世界500强企业上加大力度；培育发展外向型企业，支持外贸龙头企业参与国际市场竞争，积极发展服务贸易，进出口额年均增长20%，不断提高经济外向度。

　　———增强自主创新能力要有新举措。

　　壮大创新主体，支持企业搞好关键领域技术开发，建成国内、省内一流的石油天然气化工、乳业、新材料等研发中心。搭建创新平台，继续引进大学和科研院所，加强科技孵化器、中试基地等载体建设，加快科技成果转化应用。激发创新动力，引进培养高层次人才，加大科技投入和奖励扶持力度，研发投入占经济总量比重达到2%以上。

　　———构建资源节约型和环境友好型社会要有新进展。

　　坚持产业开发与资源节约并重、经济发展与生态保护并重，促进经济、社会和环境协调发展。大力发展循环经济，培育循环经济型企业，建设生态工业园区。推进节能降耗，抓好再生能源利用，严格土地管理，逐步提高资源利用效率。倡导绿色消费，创建节约型城市。加强生态建设和环境保护，综合整治水环境，治理工业污染和大气污染，确保区域环境质量稳定，保护湿地、森林、草原，继续实施“堵西北风口”等防风治沙工程，建设生态型园林城市。

　　———维护社会和谐稳定要开创新局面。

　　以提升市民文明素质为重点，繁荣教育、卫生、文化、体育等社会事业，加强精神文明建设，提高社会文明程度；以依法行政为重点，加强民主法制建设，规范施政理政行为，维护社会公平正义；以维护社会治安为重点，严厉打击各种违法犯罪活动，妥善处理社会矛盾，保持社会安定有序；以食品药品市场整顿为重点，维护市场秩序，保护消费者权益。

　　各位代表，2007年是大庆可持续发展新跨越的起步之年，政府工作总的要求是：坚持以科学发展观统领经济社会发展全局，深入贯彻市七次党代会精神，全力落实“五个突破”的工作部署，保发展、快建设、重民生、促和谐，努力为实现大庆可持续发展新跨越奠定基础。今年主要预期经济指标是，地区生产总值增长9%，固定资产投资增长25%，城镇居民人均可支配收入和农民人均纯收入分别增长8%和6%。围绕这一思路和目标，重点抓好10个方面工作。

　　（一）加快新型工业化进程，切实转变经济增长方式。依靠科技保证石油稳产，上大项目壮大石化产业，做好园区发展地方工业。全市工业增长速度保持8%左右，地方工业增速达到35%。确保油田稳产。支持石油企业实施“油气并重、内外并举”的科学发展模式，努力寻找新油气，搞好油田精细开发，生产原油4160万吨、天然气26亿立方米。发展大项目。确保10万吨苯乙烯投产、10万吨abs开工，落实60万吨乙烯并线改造项目，争取新建45万吨聚丙烯项目立项批复，做好6.5万吨1,4丁二醇、蓝星8万吨苯乙烯前期工作，争取早日开工。继续推进地方工业“百项工程”，重点跟踪金锣10万吨大豆肽、忠旺10万吨铝深加工和50万吨cpp等大项目，全年新建续建工业项目200项。优化园区布局。以高新区为龙头，组合石化公司、石化总厂要素资源，突出高科技，发展大项目，着力打造以精细化工、农产品深加工、新材料、生物工程、电子信息为重点的东部产业聚集区。以实现西城工业园跨越发展为目标，联手油田公司、石油管理局等中直大企业，抓好让胡路“后四厂”搬迁，建好闽南、温州等园中园，努力构筑以装备制造、新型建材、日用化工为主体的西部产业聚集区。打破行政区划限制，实现统筹发展，整合轻纺、橡胶、玻璃等工业园区，加快建设南部轻工产业聚集区。用足哈大齐工业走廊政策，支持各县区发展特色园区，不断提升园区规模、质量和水平。壮大高新技术产业。发挥高新区引领地方经济作用，重点引进“大高外”立区、立市项目。做好中油集团装备制造业基地承接服务工作，加快发展先进制造业，形成新的产业优势。用好科技专项资金，支持企业、大学和科研院所，发挥人才、技术、设备优势，加强科技研发和攻关，搞好产学研联合，促进科技成果转化。倡导节能降耗。推行清洁生产，发展循环经济，引导企业和市民节约水电等各种能源。加强工业规划控制和产业衔接，推进园区化集约生产。搞好工业废物和再生资源综合利用，探索发展、节约、环境相统一的科学模式。

　　（二）发展现代农业，加快建设社会主义新农村。坚持以城市化提升农村，以工业化带动农业，以市场化富裕农民，深入推进“三通四建五保六帮”工程，促进农村经济社会全面进步。加强基础建设。建设抗旱保收田103万亩、高标准现代化示范田10万亩、绿色有机和无公害食品基地50万亩，新建农机作业合作社54个。提高畜禽良种覆盖率，完善疫病防治、技术推广等服务体系，促进畜牧业提质增效。扶持壮大龙头企业，培育奶牛、生猪、玉米、大豆等产业基地，积极发展农村合作经济组织，形成企业、基地、农户一体发展的良性机制。发展农村公共事业。建设通乡村公路500公里、通屯路500公里，完成饮水安全工程112处，新增户用沼气5000户。普及巩固农村义务教育，推进农村中小学校标准化建设，新建扩建四县和大同区职业教育学校。改造新建乡镇卫生院8所，农村新型合作医疗参合率稳定在90%左右。组织农民劳务输出。实施“四个五千”培训工程，积极打造数控机床、焊接、缝纫、机电维修等劳务品牌，在北京、大连等城市建立千人劳务基地10个以上，转移农村劳动力20万人以上。搞好村镇规划建设。完善落实好新农村建设村镇规划，加快小城镇给排水、垃圾收集和污水处理等基础设施建设，积极推广太阳能、稻草砖、轻质墙板等新技术、新材料，开展村屯环境综合治理。壮大县域经济。加快农村经济工业化进程，发展立县、立乡企业，增强县域经济实力。争取国家和省政策、资金、项目支持，引导工商和民营资本向农村投入。推进农村整村扶贫开发，完善落实包帮扶制度，使贫困农民尽快脱贫。

　　（三）繁荣发展服务业，充实丰富都市生活内涵。围绕打造龙江西部商贸、物流、旅游、信息、交通中心，整体推动服务业提档升级。整合壮大商贸业。完善东西两大城区商贸功能，搞好东安地区综合开发，支持大商、庆客隆等大型商贸企业扩张规模。培育发展连锁经营等新兴业态，推进连锁超市、便利店进社区。深入实施国家“万村千乡”市场工程，扩大商业网点乡村覆盖面。加快发展物流业。建设石油物资物流园、港桥物流园。扶持壮大农产品、轻工、建材、汽配等专业市场，提高聚集辐射能力。激活做强金融业。积极引进外资银行和保险、信托、金融租赁等非银行金融机构，鼓励金融机构发展。增强商业银行增储放贷能力，发挥各类担保公司投融资功能，加大金融业对经济发展的支持力度。丰富提升文化娱乐业。大力发展文化产业，整合提升专业文化、民间文化、群众文化，完善政策，扩大规模，提高水平。启用大剧院、博物馆、图书城、青少年科技活动中心，发挥新建文化设施服务社会的功能。积极发展餐饮娱乐、文化艺术、广播影视等产业，打造品牌店、特色街、休闲区，繁荣群众业余生活，培育城市休闲文化。精心运作旅游业。塑造“石油文化、特色温泉、生态湿地”三大旅游品牌，发挥铁人王进喜纪念馆和油田历史陈列馆服务功能，建设石油科技博物馆；搞好当奈湿地人居生态村景区建设，发展乡村特色旅游，推进龙凤等城市湿地保护性开发；整体规划大庆北部温泉生态旅游度假区，启动建设北国之春温泉城，提升旅游业发展层次和水平。培育规范中介业。重点发展法律、咨询、信息等专业中介组织，加快培育能够协助政府承担事务性工作、提供公益性服务的政务类、服务类社区民间组织。研究制定有利于各类社会组织发展的财政支持、税收优惠、人员待遇等相关配套政策，推动中介组织健康发展。

　　（四）推进体制机制创新，营造良好发展环境。深化重点领域改革，激发经济社会发展活力。稳妥推进事业单位改革。对事业单位进行科学分类，合理界定职能，搞好机关后勤、文化系统、驻外办事处等单位改革工作。逐步完善公共财政体制。优化财政支出结构，统筹预算内外资金管理，重点向农村、社保、社会事业等领域倾斜。建立财政支出绩效评价制度，确保财政资金透明合法、安全高效使用。积极搞好投资管理体制改革。强化投资、贷款项目管理，建立健全政府投资项目代建制和投资决策责任追究制，完善非政府投资项目核准制、备案制。继续深化国有资产管理体制和地方国有企业改革。发挥国资委监管职能，严格产权交易，确保国有资产保值增值。推进地方粮食、建筑企业改革，支持联谊公司重组后盘活资产、壮大发展。扎实推进公务员制度改革。依法完成行政机关公务员身份确认、职务与级别确定和工资套改工作，做好参照公务员管理的事业单位审核确认工作。建立人才引进良性机制。发挥博士后流动工作站、留学人员创业园和创业中心等载体作用，搭建人才引进和创业平台。落实人才住房、工资和创业奖励等相关政策。努力为民营经济发展创造宽松条件。坚持平等待遇、放手发展、完善服务、引导规范，重点在政策扶持、市场准入、项目审批等方面，营造有利于民营企业成长的外部环境。鼓励发展行业协会，探索实行相互融资、相互担保，解决贷款难问题。对贡献大、实力强、信誉好的企业，实行信誉贷款，扶持民营经济壮大规模、提档升级。

　　（五）扩大对外开放，提高城市经济国际化水平。围绕资本跨区域重组和生产要素合理流动，拓宽合作领域，推动对外开放进入新阶段。提升招商引资质量。继续坚持招商引资“五个转变”，搞好投资环境宣传和推介，扩大大庆对外招商吸引力；推进园区招商、专业招商、委托招商、会展招商和小分队招商，突出引进规模大、科技含量高和外商投资项目，力争引进到位资金100亿元，引进世界500强企业取得实质进展。发展开放型经济。加快出口加工区报批和内陆港选址规划，畅通对外贸易渠道。扶持石油国际工程公司、中联油大庆公司等骨干外贸企业扩大规模，支持地方外贸企业做大做强，推动华嘉电子、安博瑞等高科技项目开拓国际市场，壮大石油机械、精细化工、轻工和纺织等外向型产业。以大庆国家级火炬计划软件产业基地为依托，培育发展华拓数码等服务外包龙头企业，推动服务外包产业快速发展。加强区域联合协作。利用振兴东北老工业基地和哈大齐工业走廊的扶持政策，加强科技、人才、信息以及油气资源、现代物流、旅游开发等领域的合作，促进区域要素资源共享。鼓励支持有条件的企业赴东南亚、俄罗斯及独联体开展项目投资，组织畜牧养殖、农产品加工等企业开拓中亚市场，提高企业利用两个市场和两种资源的能力。

　　（六）推进现代都市建设，开创城市发展新局面。坚持用先进理念规划城市，优化东部、西部城区布局，规划整理南部城区，推进城区建设互动发展。加快重大项目建设。实施55个市级新建续建重点工程。抓紧完成市政府行政服务中心、教育文化中心、青少年科技活动中心等续建项目。启动机场路、南三路、远望和奔腾立交桥、让通线“平改立”等路桥工程。做好铁路西站改扩建的前期准备，组织好飞机场建设。开展城市轨道交通、奥体中心、儿童游乐园、大广高速大庆段的规划论证。科学调控房地产市场。合理布局，控制总量，重点开发三永湖周边、红旗地区和五湖商务区，加快建设完善已审批的住宅项目，引导房地产业健康发展。积极推进棚户区动迁改造。加快龙凤铁东平房区、南砖厂地区拆迁改造，做好让胡路富强村改造规划，启动主城区铁路沿线、萨尔图铁东铁西及火车站周边地区环境治理。加大生态环境建设力度。全力支持配合油田企业，启动建设从采油五厂至六厂，纵贯南北的百里绿色油田示范带。开工建设3个生态园，新建城市绿地350公顷，植树造林10万亩，建成区绿化覆盖率达到37%。治理黎明河及果午泡、东风湖、新潮湖、燕都湖等湖泡，改造扩建东城区、八百垧和乙烯污水处理厂。积极争取国家和省里政策支持，加强退耕还林、防沙治沙、湿地保护等项目建设，加快生态市建设步伐。提高城市精细化管理水平。以区为主，继续实施“四化一畅”城管工程，集中开展环境卫生和交通秩序综合整治，强化集贸市场、繁华地段、主要街路和城乡结合部等区域的联合治理，采取市场化方式运作环卫作业、城区亮化和小广告清理。扩大数字化城市管理网络覆盖范围，运用信息手段管理城市。

　　（七）发展各项社会事业，全面提高市民素质。以创建全国文明城市为统领，推动城乡社会事业全面进步，努力提高政府公共服务水平。坚持教育优先发展。均衡基础教育，发展职业教育，提升高等教育。完成靓湖学校建设，新建万宝三区小学、红旗地区和奥林国际公寓九年一贯制学校，改善市区相对薄弱学校，加大农村教育投入，保障家庭经济困难学生受教育机会。加快职业教育发展步伐，优化市区职业教育结构，加强实验实习基地建设。支持高等教育更快更好发展，研究建立高校之间、校企之间交流合作、人才共享、科技创新平台，充分发挥高校服务地方经济发展的作用。提高居民健康水平。易地新建市中医院，扩建油田总医院。规范发展社区卫生服务，不断提升服务质量和水平。加快乡镇卫生院建设，用3年时间全部完成改扩建任务。设立红十字会医疗救助专项资金，解决突发意外、急危重病等伤病者基本医疗费用。创建国家卫生城市。繁荣文化体育事业。推进和谐文化建设，发挥“书香大庆”、“大庆之冬”、“激情之夏”等载体作用，丰富群众文化生活。开展“文化村标准化建设年”活动，搞好农村文化馆、图书馆和文化活动室建设。严厉打击“黑网吧”和非法出版物，整顿规范文化市场秩序。深入开展全民健身活动，积极承办省级以上赛事。提高社会文明程度。着力建设社会主义核心价值体系，弘扬“八荣八耻”荣辱观和大庆精神、铁人精神，加强群众社会公德、职业道德和家庭美德教育，塑造大庆人崭新形象。积极开展科技、卫生、文化三下乡活动，宣传评选文明乡、文明村、文明户，培养造就新型农民。加快构建社会信用体系，努力打造诚信大庆。

　　（八）提高社会保障水平，努力改善人民生活。切实维护民利、改善民生。扩大就业和再就业。落实新一轮就业再就业政策，新增就业2.5万人，开发公益性岗位1200个，城镇登记失业率控制在4.5%以内。发展非正规劳动就业，探索市场化创业培训模式，完善创业培训与小额担保贷款联动机制，加强大中专毕业生和复转军人就业指导和服务。健全社会保障体系。扩大养老、失业、医疗、工伤、生育保险覆盖面，逐步规范建筑行业、个体私营养老保险参保缴费，完善城镇职工基本医疗保险，做好机关、事业单位工伤、生育保险启动工作。完善农村新型合作医疗制度，全面推行农村最低生活保障制度，将符合条件的城市贫困人口全部纳入低保范围，建立覆盖城乡的低保体系。强化和谐社区建设。提升社区功能，改善硬件设施，满足社区群众精神文化需求。完善家政、福利、卫生等10大服务体系，拓展“96760”社区热线服务内容，健全覆盖全市的社区居民服务网络。努力为民多办实事。建设经济适用住房，改造老居住区环境，完成主城区已建住宅天然气入户工程。落实农村中小学生“两免一补”政策，开展“一帮一”、“希望工程”等救助活动，建设第二福利院和流浪儿童救助中心。整顿市场经济秩序，开展食品药品安全专项整治，坚决打击制假售假等违法犯罪活动。加强工商市场管理，对自发形成的早晚市进行规范，方便群众生活。

　　（九）加强地企联合协作，推进区域经济协调发展。坚持“责任共担、发展共促、稳定共抓、城乡共建”方针，自觉维护地企合作的大好局面。全力为大企业搞好服务。整治油田周边治安秩序，清理占压油气管线和生产区内违法违章建筑，营造良好生产环境。支持存续企业深化改革，建立现代企业制度，实现多元发展。帮助企业落实好新上项目的规划、环保、审批等建设条件，确保适时开工建设。积极为下岗失业人员创造就业岗位，搞好矛盾纠纷排查调处，协助企业做好安全生产工作，维护企业稳定。推进地企合作向纵深发展。共同搞好生态环境治理和棚户区改造，加快路桥等城市重大基础设施建设。联合开发“四低”小油田和气田，研究搞好化工原料吃配，共同利用好油气和石化资源。借助石油石化大企业资产、技术和人才等优势，联合开拓市场、引进资金、建设项目。合力落实国家支持资源型城市可持续发展的各项政策。构建新型地企协调机制。深化高层协调，建立重大事项通报制度，做到相关政策一起定、全市大事一起谋、大局工作合力抓。搞好中层对接，明确规划、环保、土地、房产等重点部门的工作对接方式和流程，保证工作有序衔接、运转流畅。

　　（十）完善民主法制建设，努力实现社会和谐稳定。深入开展普法教育，推进依法治市，加快社会民主化、法治化进程。加强民主政治建设。自觉接受人大监督、执行人大决议，充分尊重政协政治协商、民主监督和参政议政作用，自觉支持各民主党派、工商联和工青妇等群团组织工作，认真办理人大代表建议和政协委员提案。强化社区居委会职能建设，搞好社区换届选举，提高自治水平。完善村民自治功能，保障农民对政务财务的知情权、参与权和监督权。维护群众合法权益。发挥市长热线、援助中心等维权机构作用，畅通社情民意反映渠道。促进社会公平正义，维护好妇女、儿童、老年人、残疾人合法权益。提高社会管理水平。完善突发公共事件预案和防控体系，增强公共安全应急处置能力；创新社区管理和服务机制，提高社会事务管理水平；发挥行业协会、商会等社会团体功能，形成政府负责、社会协同、群众参与的社会管理格局。加快平安大庆建设。严厉打击各类违法犯罪，完善社会治安防控体系，实施社区和农村警务战略，深入开展打击黄赌毒和“打黑除恶”专项斗争。坚持“调访一体化”，在依法治访的同时，健全利益调节机制、诉求表达机制和矛盾调处机制，统筹兼顾社会各方面利益关系。继续支持政法机关基础设施建设。加强重要危险源监控和重大事故隐患治理，杜绝重特大事故发生，确保人民群众生命财产安全。

　　各位代表，完成今年乃至今后五年全市经济社会发展目标任务，必须切实加强政府自身建设，着力提高行政管理效能，加快构建公共服务型政府。

　　要转变政府职能，建设服务政府。在继续完善经济调节、加强市场监管的同时，把政府工作的重点、政策支持的重点、财力保障的重点放在社会事业、社会管理和公共服务上，更加注重解决就业、就学、就医、社保和救助等民生问题，更加注重生态治理、环境保护等基础建设，更加注重建立健全社会矛盾调处机制和公共安全防控体系，增强政府提供公共产品的能力和水平。

　　要坚持依法行政，建设法治政府。坚持政务公开，对政府重大事项决策、重点工作进展、重要事件处置等全部公开，确保行政权力透明运行。清理规范政府和部门规范性文件，依法审定行政执法主体资格，精简行政审批项目和公用事业服务收费项目。继续推行行政执法责任制，探索完善综合执法体制，整合区域之间、部门之间和部门内部的执法资源，建立联合执法、统一执法、集中执法的新体制，提高执法效力。坚持行政问责与行政监察、审计监督相结合，严格落实行政问责制度，有责必问，有错必究。要完善工作运行机制，建设责任政府。建立部门间的联席会议制度，提高行政决策和执行效率。设立大庆市经济发展服务中心，与行政服务中心合署办公，整合入驻部门职能，一口受理、并联审批、限时办结。加强电子政务建设，完善推广网上办公办事系统。建立政府工作评价体系，发挥第三方机构和非政府组织的监督评价功能，提升政府工作绩效。坚持集体讨论、专家咨询、法律顾问、群众参与、社会公示和听证等制度，充分发扬民主，广泛集中民智，努力做到依法、民主、科学决策。

　　要注重求实务实落实，建设效能政府。重视调查研究，深入基层、群众、企业，切实解决工作推进中遇到的实际问题。强化督办检查，提高督办措施的针对性和操作性，确保大事实事和重点工作稳步推进。实行绩效考核，健全目标考核体系，开展民主测评和绩效互评，综合评价部门工作业绩。落实公务员考核制度，严格兑现奖惩，支持鼓励能谋事、干事、成事的部门和人员。

　　要加强政风和公务员队伍建设，建设廉洁政府。强化行政效能监察，对窗口单位、关键部门、实权岗位进行重点监督，严肃查处侵害企业和群众利益行为。深入开展商业贿赂治理，重点整治工程建设、土地出让、产权交易、医药购销、政府采购、资源开发和垄断行业中的违法违纪行为。认真执行廉政建设各项规定，切实增强政府工作人员拒腐防变能力。强化审计监督，严肃财经纪律，勤俭节约办事，压缩会议，减少应酬，弘扬艰苦奋斗之风。

　　　　各位代表，大庆正站在新的历史起点上。让我们在市委的正确领导下，以科学发展观为统领，传承发扬大庆精神和铁人精神，团结、带领和依靠全市各族人民，抢抓历史机遇，力攀战略新高，为大庆可持续发展实现新跨越而努力奋斗！
